# Editorial: Porcine Anti-Viral Immunity

**DOI:** 10.3389/fimmu.2020.00399

**Published:** 2020-03-06

**Authors:** Anastasia N. Vlasova, John E. Butler

**Affiliations:** ^1^Food Animal Health Research Program, CFAES, Ohio Agricultural Research and Development Center, Department of Veterinary Preventive Medicine, The Ohio State University, Wooster, OH, United States; ^2^Department of Microbiology and Immunology, Carver College of Medicine, University of Iowa, Iowa, IA, United States

**Keywords:** swine, viruses, immune response, vaccines, immune evasion, emerging

## Introduction

The swine industry is an important part of agriculture in many countries, generating over $11 billion annually in the US alone. Viral diseases in pigs pose a serious risk for swine health and significantly impact the economy of the global swine industry. Pigs also serve as zoonotic reservoirs for viruses transmittable to humans and other species, including influenza A virus, Nipah virus, and hepatitis E virus (1). These problems are made worse by globalization and industrial livestock rearing (2, 3). Unlike bacterial pathogens, antibiotics do not protect against viral infections and cannot be used to control viral outbreaks or epidemics. Since viruses can alter their genome >10^6^ times faster than their mammalian hosts (4), the latter would have succumbed to microbial infection were it not for their adaptive immune system that uses somatic gene rearrangement and mutation to counter the rapid diversification of microbes. Immediately following viral infection, neonatal survival depends on innate immunity and passive protection by lactogenic immune factors such as pathogen-specific antibodies, until an adaptive immune response can develop. Thus, intervention against these moving targets, depends on availability of effective maternal and neonatal vaccines and strict biosecurity measures.

Wide-spread porcine reproductive and respiratory syndrome virus (PRRSV) and swine influenza virus (SIV) represent major health challenges in the large US swine production systems and possibly worldwide. In the US alone, economic annual losses due to PRRSV are estimated to be 664 million U.S. dollar (USD) (5). While the exact costs associated with SIV and other endemic viruses, including rotavirus, are hard to evaluate, they are economically important due to their ubiquitous nature, which can reduce growth and increased morbidity (6). Emerging and re-emerging coronaviruses in pigs are quite often associated with diarrheal disease and high morbidity and mortality (7). The emergence of Seneca Valley virus (SVV) and increased incidence of SVV-associated vesicular disease alarmed the swine industry in several countries, including the US, Brazil, China, Thailand, and Colombia (8). Finally, a deadly swine disease, caused by African Swine Fever virus (ASFV), that has been plaguing Africa for decades, has now spread to south eastern Europe and Asia, and has severely impacted the world's largest swine industries in China (9).

In this special volume of Frontiers in Immunology, Comparative Immunology section, Lager and Buckley provide an overview of the importance of swine in the world food supply and a review of the major viral infection that threaten this species (Lager and Buckley). In introducing the topic of anti-viral immunity, we emphasize the genetic diversity of viruses, the virus life cycle and the pathology that viral infection can cause. A total of 12 articles are contained in this volume to which 86 researches have contributed.

## Newborns are More Vulnerable to Viral Diseases

Despite the advantage of internal offspring development and the provision for passive immune protection, fetuses and neonates of higher vertebrates still remain vulnerable to environmental pathogens. Piglets *in utero* are protected from eukaryotic parasites and bacteria, but are vulnerable to viruses (including parvovirus, PRRSV, and porcine circovirus 2) since they are able to cross the placental barrier (10–12). These cause pathological injury and often fetal abortion. In a number of mammals, viruses infect the thymus and can also cause hypergammaglobulinemia. Butler et al. discuss the implication of thymic atrophy and apoptosis of thymocytes and hypergammaglobulinemia with regard to the persistence of PRRSV. However, apoptosis is also a host mechanisms used to eliminating virus-infected cells (13), but can also promote spread of the virus (14). Fernandes et al. demonstrated that SVV developed a 3C protease-dependent mechanism for late apoptosis that facilitates virus release from infected cells. Thus, the role and importance of virus-induced apoptosis awaits further research.

Since the adaptive immune system of fetal piglets is underdeveloped, the innate immune system is very important. Schäfer et al., emphasize its importance with the specific focus on porcine invariant natural killer T cells (iNKT) and their role in the pathogenesis of ASF and SI. They discuss iNKT age-dependence, levels and distribution in relationship to various porcine viral infections. One of the first host responses to viral infection is the production of interferons, which are needed to drive other elements of innate immunity and adaptive immunity. Likai et al. show how a porcine deltacoronavirus escapes from the immune system by suppressing IFN-α production, while Shi et al. describe a novel immune evasive mechanism that depends on PRRSV non-structural protein 1a which antagonizes TBK1-IRF3-IFN signaling. While the initial antibody response depends on broadly specific natural IgM antibodies, effective anti-viral immunity depends on an adaptive immune response that delivers IgG and IgA antibodies. Since adaptive immune responses depend on stimulation through the innate immune system, viruses that impair or interfere with the innate immune response, also impair adaptive immunity. Nedumpun et al. discuss how PRRSV-induced IL-1Ra downregulates innate immune responses, T lymphocyte differentiation and proliferation.

Piglets, unlike humans, but like the offspring of other artiodactyls and perissodactyls (horses), are especially dependent on lactogenic immunity since there is no transplacental transfer of passive antibodies in this group of mammals. Thus, providing ways to deliver anti-viral antibodies via colostrum/ milk is important. This topic is the focus of studies by Langel et al..

## Anti-Viral Immunity and Vaccine Development

Antibodies can intercept and neutralize a virus before it infects additional cells and can prevent further infection by blocking the viral receptor; these are called virus neutralizing (VN) antibodies. Naturally, a critical step in viral vaccine design is the identification of viral epitopes targeted by effective VN antibodies. This become increasingly important in the case of PRRSV in which effective VN antibodies are slow to appear and current vaccines are of questionable efficacy. This is also an ongoing challenge in the case of ASFV which is rapidly spreading and for which no vaccine exists. Goldeck et al. describe a novel B cell cloning procedure to identify these epitopes on several strains of PRRSV. While VN antibodies can block or delay infection, the ultimate elimination of a virus is to kill the cells in which the virus replicates which is the job of cytotoxic T cells. Portions of the virus are displayed on infected cells. These are called a T cell epitope, i.e., a molecular structure that binds to the T cell receptor (TCR). In this volume, Pan et al. describe their efforts to identify such epitopes for their potential use in stimulating expansion of the cytotoxic T cells that recognize them.

Viral vaccines take various forms, the simplest being the use of killed virus. A more tedious procedure is to use only parts of the virus as the vaccine (subunit vaccines) that target the immune response to those viral epitopes that elicit VN antibodies. Killed viruses and subunit vaccines typically generate immediate responses that wane faster, making it imperative for long-lived mammals like humans, to receive booster vaccinations. A second approach to vaccine development is use of live attenuated virus that has been genetically modified or cell culture adapted and cannot produce a disease in the host but can still replicate. These are often more likely to stimulate virus-specific cytotoxic T cells and to induce longer term immunity. Sharma et al. developed a recombinant SVV strain using reverse genetics and tested its immunogenicity and protective efficacy in pigs. Toman et al. provide comparative data on four vaccines for PRRSV, two killed and two modified live.

We believe that veterinary immunovirology should place more emphasis on how each viral pathogen effects and/or avoids the host immune system and on the identification of viral epitopes that are effective targets of VN antibodies and T cell epitopes that can promote the action of cytotoxic T cells.

## Author Contributions

JB wrote the first draft and then both co-authors contributed equally to the final draft.

### Conflict of Interest

The authors declare that the research was conducted in the absence of any commercial or financial relationships that could be construed as a potential conflict of interest.
